# Transition mechanism for a periodic bar-and-joint framework with limited degrees of freedom controlled by uniaxial load and internal stiffness

**DOI:** 10.1098/rsos.180139

**Published:** 2018-06-13

**Authors:** H. Tanaka, K. Hamada, Y. Shibutani

**Affiliations:** 1 Department of Mechanical Engineering, Osaka University, 2-1 Yamadaoka, Suita, Osaka 565-0871, Japan; 2 Nanotechnology Program, Vietnam Japan University, Luu Huu Phuoc Street, My Dinh 1 Ward, Nam Tu Liem District, Ha Noi, Viet Nam

**Keywords:** transition mechanism, nonlinear elasticity, periodic bar-and-joint framework, structural stability

## Abstract

A specific periodic bar-and-joint framework with limited degrees of freedom is shown to have a transition mechanism when subjected to an external force. The static nonlinear elasticity of this framework under a uniaxial load is modelled with the two angular variables specifying the rotation and distortion of the linked square components. Numerically exploring the equilibrium paths then reveals a transition state of the structure at a critical value of the internal stiffness. A simplified formulation of the model with weak nonlinear terms yields an exact solution of its transition state. Load–displacement behaviour and stability for the two systems with or without approximation are analysed and compared.

## 1. Introduction

Although numerous periodic and symmetric structures have been designed in the past, they remain of great interest to the scientific community [1–3]. The topic appears in a wide range of fields from geometry to crystallography to engineering, and indeed the mechanical behaviours of many of these structures remain unexplored. Some examples of such behaviours include auxeticity in materials of negative Poisson’s ratio [4–9], origami-based folding and deployment [10–14], and deformability of hierarchically arranged structures [15–17], in addition to the fundamental mechanical properties of rigidity and flexibility [18–22].

In recent years, a variety of artificial microstructures with multi-functionality have been extensively developed by incorporating specific geometric features [23,24]. In a similar strategy regarding hierarchical biomaterials provided by natural selection [25–28], for lightweight solid constructions, in particular, there are the two key trends in mechanical design. One is the optimized design of material-selected structures using dissimilar materials by means of bonding technology [29,30]; the other is developing structure-selected materials, which is made possible by advances in microstructure fabrication at the micro/nanometre scale [31–35]. Indeed, further active fusion between the fields of materials science and structural mechanics is required to create new mechanical designs that implement the two hybrid approaches.

Classical structural mechanics has established elastic stability for conservative component systems [36,37]. The nonlinear elastic behaviour of structures such as buckling and the analogous unstable phenomena are sufficiently well understood analytically and numerically. Most behaviours can be classified according to some representative component system, for example column buckling and snap-through and snap-back buckling [38]. Subsequently, analyses and experiments with periodic and symmetric structures such as honeycombs have revealed both linear and nonlinear mechanical responses within microstructures [3,18,19,39–45]. This series of studies has been linked recently to the development of novel structural systems with functionalities induced by altered morphology and elasticity [46–53].

In this context, our previous study proposed a cellular structure with unique connectivity and eightfold rotational symmetry [54]. Depending on the direction of uniaxial loading, this structural system undergoes two types of kinematic transformations, diamond- and square-patterned unit cells [55], here termed Pattern-D and Pattern-S, respectively. Controlling the point of loading enables the structure to switch between these two motions. Taking cell-to-cell contact into account, bi-stiffness develops through the strong anisotropy of the square cells. We further extend this structure to create a periodic structure by joining and inserting linear springs between adjacent unit cells. Finite-element analyses demonstrated that the periodic structure also has a similar transition mechanism [56]. In this instance, the deflection of each cell plays a major role in the transition from one motion to another. Specifically, if *k* is the constant of the cell-binding springs and *EI*/ℓ^3^ is the bending stiffness of cells, the relative spring constant, $\tilde{k}\equiv k\ell^{3}/EI$, determines whether the structure undergoes Pattern-D or -S. However, the transition state of such cellular structures is often difficult to understand in detail because the mathematical description of bending deformation is complex and uncountable deformation patterns made up of multiple unit cells potentially emerge.

To clarify the transition mechanism from the viewpoint not only of structural kinematics but also of mathematical insight, we focus on modelling a specific bar-and-joint framework that replaces flexible cells with linked squares. This process of modelling imposes strict restrictions on the allowed complex deformations of a cellular structure such as non-affine deformation patterns of a periodic lattice structure [57]. As a result, the periodic bar-and-joint framework only involves an affine transformation represented by a single unit cell. We further formulate the reduced model for a single-unit-cell analysis with reflection symmetry about the vertical axis along the direction of uniaxial loading by excluding a shear deformation of the entire repetitive structure, which enables us to construct non-trivial particular solutions of the transition mechanism under pure compression. Although the proposed bar-and-joint framework has specific geometry and the transition mechanism obtained is a highly constrained example, the main idea is that this abstract representation might provide an original perspective on nonlinear elastic phenomena in solid-state matter.

This work is organized as follows. In §2, we propose a periodic bar-and-joint framework linked with two types of linear and rotational springs. Under the assumption of axially symmetric deformation under uniaxial loading, we formulate the total potential energy of the dimensionless structural system with two angular variables. Solving the balanced equation for the two angular variables, we numerically analyse the equilibrium paths with regard to the internal stiffness of the system to its critical value associated with the transition state. In §3, we simplify the structural system by introducing approximations for the weak nonlinear terms and mathematically derive the critical point for the corresponding transition in the simplified system. Using the acquired information, we derive a load–displacement relation for the primary and secondary paths in the transition state, and discuss system stability. We present a summary and conclusion in §4.

## 2. Structural system

### 2.1. Framework setting

**Figure 1.**
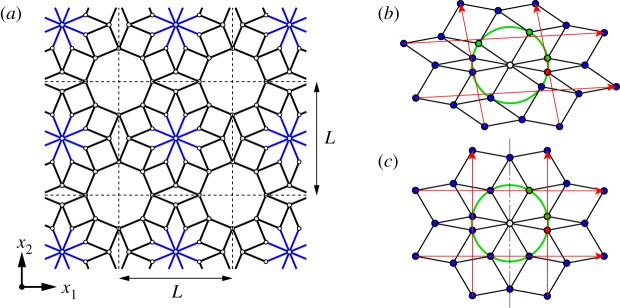
(*a*) Proposed periodic bar-and-joint framework; (*b*) unit cell subjected to a transformation with three rotational degrees of freedom; (*c*) unit cell subjected to a transformation with two rotational degrees of freedom that retains reflection symmetry about the vertical axis through the centre.

A bar-and-joint framework is illustrated in figure 1*a*. This framework has been assembled using a single type of unit cell of length *L* in a two-dimensional orthogonal array. In the initial configuration, each unit cell is composed of two types of inextensible rigid struts (bars). A blue straight bar of twice the length of a black bar is pivotally connected (joined) at each end; the midpoints of the four blue bars are pinned to each other at the centre of each unit cell. The black straight bar is also joined on each end; four black bars form a square linkage. Eight square linkages are joined to encircle the four blue bars; the outer vertices of the squares are linked with those in the adjacent cells.

The proposed periodic structure transforms under three rotational degrees of freedom. In the following, details of the mechanism are explained in a visual way based on geometry [58,59]. The green circle in figure 1*b* shows the possible range of rotation of the bars pinned at the centre. If a joint, indicated in red, is fixed on the green circle, three other joints, indicated in green, can move to any place on the circle so that the four bars corresponding to these four joints are uniquely positioned. Then, the eight rhombi extending from the centre and the surrounding eight rhombi are sequentially determined. The transformed unit cell has central symmetry, and hence the pair of parallel red vectors between specific vertices generate the periodic lattice. Considering that this periodic assembly needs the connections of four outer vertices per unit cell, the structure is over-constrained and thus the three parameters represented intrinsically by the green joints determine the entire periodic bar-and-joint framework.

From figure 1*b*, the unit cell undergoes shear deformation as a whole, which is an undesirable motion as this study focuses on pure uniaxial compression. For example, assembling the unit cells to form a structure with large cross-sectional area against compression, we can reduce one of the degrees of freedom. This corresponds to imposing a reflection symmetry about the vertical line through the centre of the unit cell, which is equivalent to a unit cell being transformed under an operation with two rotational degrees of freedom, determined by the positions of the two green joints in figure 1*c*. During compression, the two red vectors of the lattice are perpendicular to each other. We herein define a horizontal axis *x*_1_ through the centre and the midpoint between the red joint and the adjacent green joint. Then, the vertical axis *x*_2_ is perpendicular to *x*_1_ at the origin. When the orthogonal coordinates (*x*_1_,*x*_2_) are fixed in a space, the transformed structural unit under compression holds reflection symmetry with respect to the *x*_1_- and *x*_2_-axes because of its own central symmetry. Note that the illustration mentioned above will appear in §(b).

**Figure 2.**
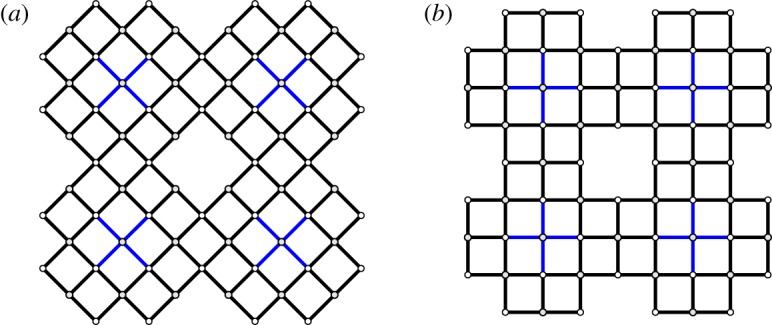
Characteristic mechanism to obtain the bar-and-joint framework by rotating squares: (*a*) complete Pattern-D and (*b*) complete Pattern-S using 2×2 unit cells.

Assuming that each of the square linkages of four equal sides is not distorted, the periodic structure allows only the squares to rotate in the characteristic mechanism. According to their coordinated motion, the structural system transforms into square cells tilted at a 45^°^ angle (diamond cells) as in Pattern-D or into square cells aligned along the initial periodic directions as in Pattern-S [55]; see figure 2*a*,*b*, respectively.

### 2.2. Formulation of the total potential energy

**Figure 3.**
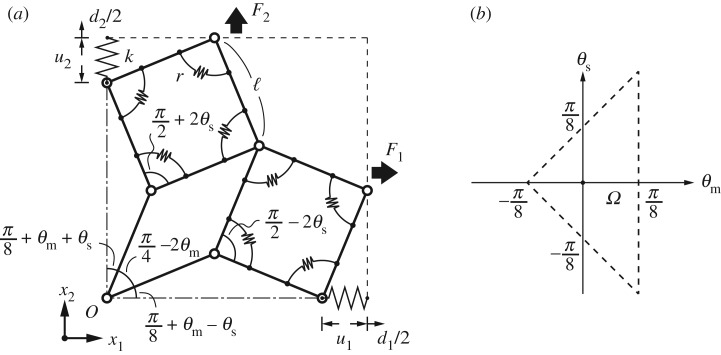
(*a*) Bar-and-joint framework of the proposed unit cell with the first of four structural elements. The structure is linked by linear and rotational springs; (*b*) the domain *Ω* of *θ*_m_ and *θ*_s_, where the dashed lines indicate contacts between components.

Consider a unit cell transformed under an operation with two rotational degrees of freedom (figure 1*c*). Imposing the vertical axis of symmetry on the deformation mechanism constrains the analytical model into using four central structure elements (figure 3*a*), comprising identical bars of length ℓ joined at each end. In this structural system, two types of independent rotational motion exist and we describe their motions with two angular variables, denoted as *θ*_m_,*θ*_s_∈(−*π*,*π*), which are hereafter referred to as the *two state angles*; the former describes the rotational paths of Pattern-D (*θ*_m_>0) or Pattern-S (*θ*_m_<0), and the latter describes the rhombic distortion of the linked squares. Taking the bar-to-bar contact into account, we find the parameter space of *θ*_m_ and *θ*_s_ to be constrained to the domain

2.1 $$\Omega =\left\{(\theta_{m},\theta_{s})|\theta_{m}<\frac{\pi }{8},\theta_{m}\pm \theta_{s}>-\frac{\pi }{8}\right\},$$

which is depicted in figure 3*b*. Let $F={\left(F_{1}\;F_{2}\right)}^{T}\in R^{2}$ be the force acting on a unit cell in the *x*_1_- and *x*_2_-axial directions (the tensile force being positive). Applying an arbitrary force ***F***, the associated displacement vector is then

2.2 $$\begin{array}{rl} d & =\left(\begin{array}{c} d_{1}\\ d_{2} \end{array}\right)\\ & =\left(\begin{array}{c} 2\ell \left\{\cos\left(\frac{\pi }{8}+\theta_{m}-\theta_{s}\right)+\sin\left(\frac{\pi }{8}+\theta_{m}+\theta_{s}\right)-2\cos\frac{\pi }{8}-\sin\frac{\pi }{8}\right\}\\ 2\ell \left\{\cos\left(\frac{\pi }{8}+\theta_{m}+\theta_{s}\right)+\sin\left(\frac{\pi }{8}+\theta_{m}-\theta_{s}\right)-2\cos\frac{\pi }{8}-\sin\frac{\pi }{8}\right\} \end{array}\right). \end{array}$$

The magnitude of the stiffness of the overall system is determined by the two types of internal stiffness, i.e. the two constants of the linear half-length spring and the rotational spring, $k,r\in R_{+}$; here $R_{+}$ denotes the set of positive reals. With the above-defined parameters, the total potential energy of the system $\Pi :R^{2}\times \Omega \times R_{+}^{2}\to R$ comprises three terms

2.3 $$\Pi =U_{k}+U_{r}-W,$$

where *U*_*k*_, *U*_*r*_ and *W* denote the potential energy for the two linear springs and 32 rotational springs in the unit cell and the work done by ***F***, respectively, each of which is expressed by

2.4 $$U_{k}=2\cdot \frac{1}{2}ku^{T}u,\;U_{r}=32\cdot \frac{1}{2}r{\left(2\theta_{s}\right)}^{2}\;\mathrm{and}\;W=F^{T}d,$$

where ***u*** is the elongation vector of the linear springs; that is,

2.5 $$u=\left(\begin{array}{c} u_{1}\\ u_{2} \end{array}\right)=\left(\begin{array}{c} \ell \left\{\sin\left(\frac{\pi }{8}+\theta_{m}+\theta_{s}\right)-\sin\frac{\pi }{8}\right\}\\ \ell \left\{\sin\left(\frac{\pi }{8}+\theta_{m}-\theta_{s}\right)-\sin\frac{\pi }{8}\right\} \end{array}\right).$$

We then introduce the dimensionless parameters,

2.6 $$\tilde{\Pi }=\frac{\Pi }{32r},\;\tilde{k}=\frac{k\ell^{2}}{16r},\;\tilde{F}=\frac{\ell }{32r}F,\;\tilde{d}=\frac{1}{\ell }d\;\mathrm{and}\;\tilde{u}=\frac{1}{\ell }u.$$

For convenience, the two state angles are converted to

2.7 $$\phi =\theta_{m}+\theta_{s}\;\mathrm{and}\;\psi =\theta_{m}-\theta_{s},$$

giving inverse relations

2.8 $$\theta_{m}=\frac{\phi +\psi }{2}\;\mathrm{and}\;\theta_{s}=\frac{\phi -\psi }{2}.$$

From equation (2.1), the parameter domain for *ϕ* and *ψ* is

2.9 $$\Omega^{*}=\left\{(\phi ,\psi )|\phi >-\frac{\pi }{8},\psi >-\frac{\pi }{8},\phi +\psi <\frac{\pi }{4}\right\}.$$

Considering the uniaxial loading in only the *x*_2_-direction, i.e. ${\tilde{F}}_{1}=0$, the total potential energy of the dimensionless system, which defines a mapping $\tilde{\Pi }:R\times \Omega^{*}\times R_{+}\to R$, can be formulated with equations (2.3)–(2.7) as follows:

2.10 $$\tilde{\Pi }=\frac{1}{2}\tilde{k}{\tilde{u}}^{T}\tilde{u}+\frac{1}{2}{\left(\phi -\psi \right)}^{2}-{\tilde{F}}_{2}{\tilde{d}}_{2},$$

where

2.11 $${\tilde{d}}_{2}=4\cos\left(\frac{\pi }{8}+\phi \right)+2\sin\left(\frac{\pi }{8}+\psi \right)-4\cos\frac{\pi }{8}-2\sin\frac{\pi }{8}$$

and

2.12 $${\tilde{u}}_{1}=\sin\left(\frac{\pi }{8}+\phi \right)-\sin\frac{\pi }{8}\;\mathrm{and}\;{\tilde{u}}_{2}=\sin\left(\frac{\pi }{8}+\psi \right)-\sin\frac{\pi }{8}.$$

### 2.3. Equilibrium path as a function of the two state angles

Assuming that an internal stiffness $\tilde{k}$ is present, let us consider the first variation of equation (2.10) with respect to *ϕ* and *ψ*, that is,

2.13 $$\begin{array}{rl} \delta \tilde{\Pi } & =\tilde{\Pi }(\phi +\delta \phi ,\psi +\delta \psi )-\tilde{\Pi }(\phi ,\psi )\\ & ≃\frac{\partial \tilde{\Pi }}{\partial \phi }\delta \phi +\frac{\partial \tilde{\Pi }}{\partial \psi }\delta \psi . \end{array}$$

Based on the principle of stationary total potential energy ($\delta \tilde{\Pi }=0$), we obtain the following system of balanced equations:

2.14 $$\begin{array}{rl} & \frac{\partial \tilde{\Pi }}{\partial \phi }=\frac{\partial f}{\partial \phi }-{\tilde{F}}_{2}\frac{\partial {\tilde{d}}_{2}}{\partial \phi }=0\\ \;\text{and}\; & \frac{\partial \tilde{\Pi }}{\partial \psi }=\frac{\partial f}{\partial \psi }-{\tilde{F}}_{2}\frac{\partial {\tilde{d}}_{2}}{\partial \psi }=0, \end{array}\}$$

where we have introduced the function $f=\frac{1}{2}\tilde{k}{\tilde{u}}^{T}\tilde{u}+\frac{1}{2}{\left(\phi -\psi \right)}^{2}$. Eliminating ${\tilde{F}}_{2}$ from equation (2.14) gives

2.15 $$\frac{\partial f}{\partial \phi }\frac{\partial \phi }{\partial {\tilde{d}}_{2}}-\frac{\partial f}{\partial \psi }\frac{\partial \psi }{\partial {\tilde{d}}_{2}}=0.$$

For each derivative in equation (2.15), we find

2.16 $$\begin{array}{rl} \frac{\partial f}{\partial \phi } & =\tilde{k}S_{\phi }+(\phi -\psi )\;\mathrm{and}\;\frac{\partial f}{\partial \psi }=\tilde{k}S_{\psi }-(\phi -\psi ), \end{array}$$

2.17 $$\begin{array}{rl} & \frac{\partial {\tilde{d}}_{2}}{\partial \phi }=-4\sin\left(\frac{\pi }{8}+\phi \right)\;\mathrm{and}\;\frac{\partial {\tilde{d}}_{2}}{\partial \psi }=2\cos\left(\frac{\pi }{8}+\psi \right), \end{array}$$

where

2.18 $$\begin{array}{rl} S_{\phi } & ={\tilde{u}}_{1}\frac{\partial {\tilde{u}}_{1}}{\partial \phi }=\frac{1}{2}\left\{\sin\left(\frac{\pi }{4}+2\phi \right)-\sin\left(\frac{\pi }{4}+\phi \right)+\sin\phi \right\}\\ \;\text{and}\;S_{\psi } & ={\tilde{u}}_{2}\frac{\partial {\tilde{u}}_{2}}{\partial \psi }=\frac{1}{2}\left\{\sin\left(\frac{\pi }{4}+2\psi \right)-\sin\left(\frac{\pi }{4}+\psi \right)+\sin\psi \right\}. \end{array}\}$$

Let $g(\phi ,\psi ,\tilde{k}):\Omega^{*}\times R_{+}\to R$ be defined as the LHS of equation (2.15); then *g* becomes infinite when the denominator $\partial {\tilde{d}}_{2}/\partial \phi$ or $\partial {\tilde{d}}_{2}/\partial \psi$ is equal to zero. To avoid this type of singularity, we introduce the function $g^{*}(\phi ,\psi ,\tilde{k})$, defined as

2.19 $$g^{*}=\frac{1}{2}\;\frac{\partial {\tilde{d}}_{2}}{\partial \phi }\frac{\partial {\tilde{d}}_{2}}{\partial \psi }g=\frac{1}{2}\left(\frac{\partial f}{\partial \phi }\frac{\partial {\tilde{d}}_{2}}{\partial \psi }-\frac{\partial f}{\partial \psi }\frac{\partial {\tilde{d}}_{2}}{\partial \phi }\right).$$

Substituting equations (2.16) and (2.17) into equation (2.19), we obtain

2.20 $$g^{*}=\tilde{k}\left\{S_{\phi }\cos\left(\frac{\pi }{8}+\psi \right)+2S_{\psi }\sin\left(\frac{\pi }{8}+\phi \right)\right\}+(\phi -\psi )\left\{-2\sin\left(\frac{\pi }{8}+\phi \right)+\cos\left(\frac{\pi }{8}+\psi \right)\right\}.$$

Investigating the equilibrium path of the structural system is reduced to solving the following problem: find (*ϕ*,*ψ*)∈Ω* such that $g^{*}(\phi ,\psi ,\tilde{k})=0$ for a fixed $\tilde{k}\in R_{+}$.

**Figure 4.**
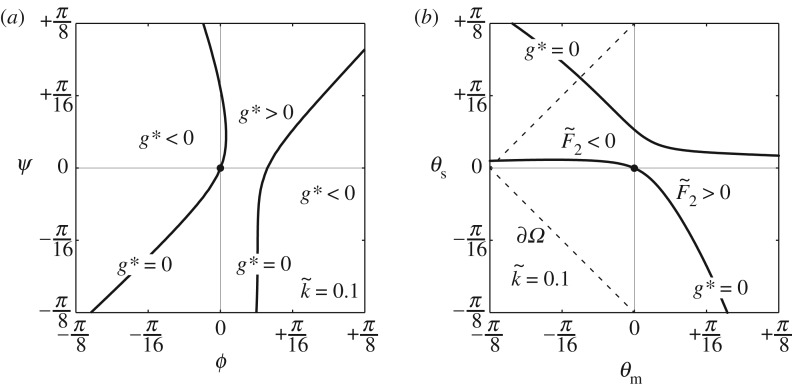
Calculated equilibrium paths of the structural system for $\tilde{k}=0.1$: (*a*) (*ϕ*,*ψ*) and (*b*) (*θ*_m_,*θ*_s_).

Figure 4*a* shows the state diagram of (*ϕ*,*ψ*) for $\tilde{k}=0.1$, derived from the procedure given above; figure 4*b* corresponds to its conversion to (*θ*_m_,*θ*_s_) using equation (2.8). In figure 4*b*, the bold curve through the origin is the equilibrium path from the initial configuration; under tension (${\tilde{F}}_{2}>0$), *θ*_m_ increases and *θ*_s_ decreases with comparable magnitude, and under compression (${\tilde{F}}_{2}<0$), *θ*_m_ decreases but *θ*_s_ remains small in value. Note that a value of ${\tilde{F}}_{2}$ can be calculated by substituting the obtained equilibrium point (*ϕ*,*ψ*) into equation (2.14). Moreover, another equilibrium path on the upper side (not through the origin) is observed. However, no physical sense can be attached to this path.

**Figure 5.**
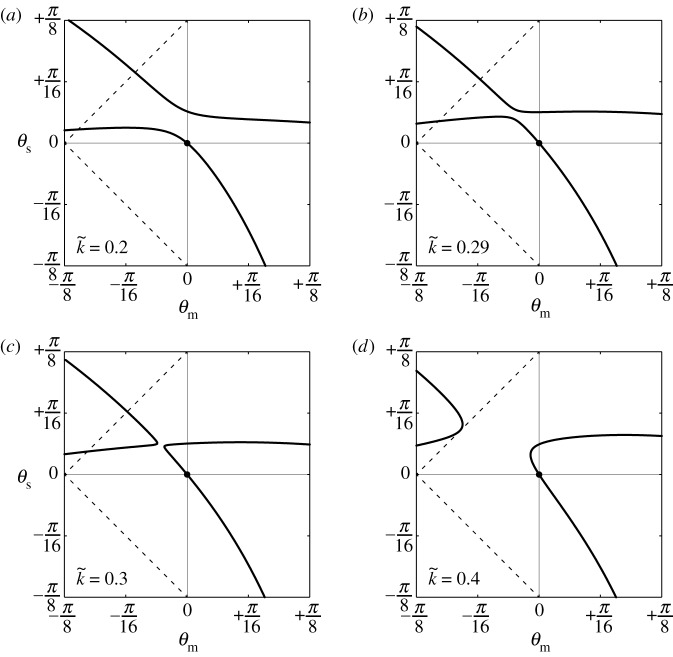
Calculated equilibrium paths (*θ*_m_,*θ*_s_) of the structural system for various $\tilde{k}$: (*a*) $\tilde{k}=0.2$, (*b*) $\tilde{k}=0.29$, (*c*) $\tilde{k}=0.3$ and (*d*) $\tilde{k}=0.4$.

Increasing $\tilde{k}$ from 0.2 to 0.4, the state diagrams of (*θ*_m_,*θ*_s_) change (figure 5*a*–*d*). Moreover, the gap between the two equilibrium paths becomes smaller as $\tilde{k}$ increases, and the primary path switches towards Pattern-D as $\tilde{k}$ passes 0.3, which means that there is at least one critical value for stiffness ${\tilde{k}}_{c}$ between 0.29 and 0.3 such that two equilibrium paths intersect. A further increase in $\tilde{k}$ widens the gap between the two paths. In contrast with this compressive behaviour, the equilibrium paths under extension remain essentially unchanged as $\tilde{k}$ increases.

**Figure 6.**
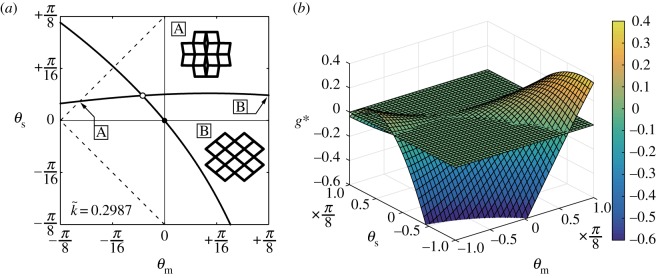
(*a*) Calculated equilibrium paths (*θ*_m_,*θ*_s_) of the structural system under a transition state with $\tilde{k}=0.2987$; the two insets A and B show the transformation shapes of a unit cell with bar-to-bar contact occurring; (*b*) three-dimensional plot of g*(*θ*_m_,*θ*_s_) given by equation (2.20) with $\tilde{k}=0.2987$.

Next, $\tilde{k}$ is tuned so as to obtain the transition state of the structural system. Figure 6*a*,*b* shows, respectively, the state diagram of (*θ*_m_,*θ*_s_) and the three-dimensional plot of the function g*(*θ*_m_,*θ*_s_) when $\tilde{k}=0.2987\equiv {\tilde{k}}_{c}$. Note that the equilibrium path through the origin bifurcates via the point of (*θ*_m_,*θ*_s_)≈(−0.0833,0.0942), which corresponds to the saddle point of g*. As a result, the transition mechanism of the system is explained as follows: the function $g^{*}(\theta_{m},\theta_{s},\tilde{k})$ necessarily passes through the origin on the hyperplane g*=0 and has a saddle point, nearby the origin. The value of the g*-function diminishes on the whole as $\tilde{k}$ increases so that g*>0 at the saddle point when $\tilde{k}<{\tilde{k}}_{c}$ or vice versa. We thus conclude that, when g* rises or falls, each of the two equilibrium paths along the level lines of g*=0 is switched from one fringe of the hyperbolic paraboloid to the other via the transition state with ${\tilde{k}}_{c}$ (figures 5*b*,*c* and 6*a*).

We also investigate the equilibrium path of the structural system for various $\tilde{k}$ under a prescribed external force ${\tilde{F}}_{2}$. As for the results obtained above, there is one critical load ${\tilde{F}}_{2c}$ such that the structural system exhibits a transition state. The details are described in appendix A.

## 3. Simplified system with weak nonlinearity

### 3.1. Formulation and preliminary numerical survey

The weak nonlinear form of the total potential energy in equations (2.10)–(2.12) is expressible in the form

3.1 $$\tilde{\Pi }≃\frac{1}{2}\kappa (\phi^{2}+\psi^{2})+\frac{1}{2}{\left(\phi -\psi \right)}^{2}-{\tilde{F}}_{2}(-2p\phi^{2}-4q\phi -q\psi^{2}+2p\psi ),$$

where $\kappa =\tilde{k}p^{2}$, p=cos⁡(π/8) and q=sin⁡(π/8); clearly, *p*^2^+*q*^2^=1. For the derivation of equation (3.1), we used $\cos\alpha ≃1-\frac{1}{2}\alpha^{2}$ and sin⁡α≃α. The simplified system (3.1) is far from a physical system, but its mathematical representation is, nonetheless, able to provide a simple understanding of the transition mechanism observed in the previous section.

The partial derivatives of terms of equation (3.1) with respect to *ϕ* and *ψ* yield

3.2 $$\frac{\partial f}{\partial \phi }=\kappa \phi +(\phi -\psi ),\;\frac{\partial f}{\partial \psi }=\kappa \psi -(\phi -\psi )$$

and

3.3 $$\frac{\partial {\tilde{d}}_{2}}{\partial \phi }=-4p\phi -4q,\;\frac{\partial {\tilde{d}}_{2}}{\partial \psi }=-2q\psi +2p.$$

Substituting equations (3.2) and (3.3) into equation (2.19) gives

3.4 $$g^{*}=\kappa \{(2p-q)\phi \psi +p\phi +2q\psi \}-(\phi -\psi )(2p\phi +q\psi -p+2q).$$

**Figure 7.**
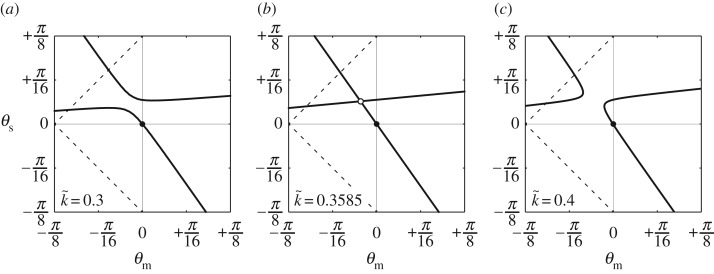
Calculated equilibrium paths (*θ*_m_,*θ*_s_) of the simplified system with various $\tilde{k}$: (*a*) $\tilde{k}=0.3$, (*b*) $\tilde{k}=0.3585$ and (*c*) $\tilde{k}=0.4$.

With the same procedure mentioned in §(c), we explored the quadric surface of g* in equation (3.4) for a fixed $\tilde{k}$ and we found the transition behaviour of the simplified system, which was similar to that of the structural system without approximation (figure 7*a*–*c*). Remarkably, the simplified system seems to have straight equilibrium paths under the transition state with ${\tilde{k}}_{c}=0.3585$. In equation (3.4), g*=0 is an implicit function, termed a conic section, the formula of which can be expanded as *aϕ*^2^+*bϕψ*+*cψ*^2^+*dϕ*+*eψ*=0. In this case, the two branches in figure 7*a* or *c* are categorized as hyperbolae [60] because

3.5 $$b^{2}-4ac={\left(\kappa +1\right)}^{2}{\left(2p-q\right)}^{2}+8pq>{\left(2p+q\right)}^{2}\;\mathrm{for}\;\kappa \in R_{+}$$

and the two lines in figure 7*b* might be regarded as a degenerate conic [61].

### 3.2. Mathematical descriptions of the transition state

Based on these preliminary results, we next analyse the transition behaviour of the simplified system (3.1). The necessary condition that the system under a transition state has a singular point is given by

3.6 $$g^{*}=0\;\mathrm{and}\;\frac{\partial g^{*}}{\partial \phi }=\frac{\partial g^{*}}{\partial \psi }=0.$$

Solving the simultaneous equations (3.6), the three types of solutions are determined as follows:

3.7 $$\begin{array}{rl} & \kappa_{c}=0,\;\phi =\frac{p-2q}{2p+q},\;\psi =\frac{p-2q}{2p+q}, \end{array}$$

3.8 $$\begin{array}{rl} & \kappa_{c}=-2,\;\phi =-\frac{p+2q}{2p+q},\;\psi =\frac{p+2q}{2p+q}, \end{array}$$

3.9 $$\begin{array}{rl} & \begin{array}{rl} \kappa_{c} & =\frac{p-2q}{pq(2p-q)}\\ \;\text{and}\;\phi & =-\frac{-p^{2}q^{2}+4q^{4}}{2p^{4}-p^{3}q+4pq^{3}-2q^{4}},\;\psi =-\frac{p^{4}-4p^{2}q^{2}}{2p^{4}-p^{3}q+4pq^{3}-2q^{4}}. \end{array}\} \end{array}$$

In equations (3.7) and (3.8), $\tilde{k}\in \;R_{+}$. Hence, equation (3.9) is the sole solution for the critical point the system has. Considering the following equation:

3.10 $$G=\frac{\partial^{2}g^{*}}{\partial \phi^{2}}\frac{\partial^{2}g^{*}}{\psi^{2}}-{\left(\frac{\partial^{2}g^{*}}{\partial \phi \partial \psi }\right)}^{2}=-8pq-{\left(\kappa +1\right)}^{2}{\left(2p-q\right)}^{2},$$

the sufficient condition that g* in equation (3.4) has a saddle point is satisfied because *G*<0 for ∀*κ*. Therefore, g* is a hyperbolic paraboloid and the critical point is consistent with a saddle point of g* at *κ*_c_.

The other expression in equation (3.9) is

3.11 $$\begin{array}{rl} {\tilde{k}}_{c} & =\frac{p-2q}{p^{3}q(2p-q)}=\frac{{\left(1+\tan^{2}(\pi /8)\right)}^{2}(1-2\tan(\pi /8))}{\tan(\pi /8)(2-\tan(\pi /8))}\approx 0.3585, \end{array}$$

3.12 $$\begin{array}{rl} \theta_{m} & =\frac{1}{2}\frac{\left(-p^{2}+q^{2})(p^{2}-4q^{2}\right)}{\left(2p-q)(p^{3}+2q^{3}\right)}=-\frac{1}{2}\frac{\left(1-\tan^{2}(\pi /8))(1-4\tan^{2}(\pi /8)\right)}{\left(2-\tan(\pi /8))(1+2\tan^{3}(\pi /8)\right)}\approx -0.0717 \end{array}$$

3.13 $$\begin{array}{rl} \;\text{and}\;\theta_{s} & =\frac{1}{2}\frac{\left(p^{2}+q^{2})(p^{2}-4q^{2}\right)}{\left(2p-q)(p^{3}+2q^{3}\right)}=\frac{1}{2}\frac{\left(1+\tan^{2}(\pi /8))(1-4\tan^{2}(\pi /8)\right)}{\left(2-\tan(\pi /8))(1+2\tan^{3}(\pi /8)\right)}\approx 0.1010. \end{array}$$

From equations (3.12) and (3.13), we have the following relation:

3.14 $$\theta_{m}+(p^{2}-q^{2})\theta_{s}=0.$$

Substituting *κ*_c_ of equation (3.9) and equation (2.7) into equation (3.4), g*(*θ*_m_,*θ*_s_) becomes

3.15 $$\begin{array}{rl} g^{*} & =\frac{p-2q}{pq}\theta_{m}^{2}-\left\{\frac{p-2q}{pq}+2(2p-q)\right\}\theta_{s}^{2}-2(2p+q)\theta_{m}\theta_{s}\\ & \;+\frac{\left(p-2q)(p+2q\right)}{pq(2p-q)}\theta_{m}+\left\{\frac{{\left(p-2q\right)}^{2}}{pq(2p-q)}+2(p-2q)\right\}\theta_{s}. \end{array}$$

Linking equation (3.14) with the two linear equilibrium paths shown in figure 7*b*, the g*-function under the transition state is factored by

3.16 $$g^{*}=b_{0}\{\theta_{m}+(p^{2}-q^{2})\theta_{s}\}(\theta_{m}+b_{1}\theta_{s}+b_{2}).$$

In comparing the coefficients of equations (3.15) and (3.16), we find

3.17 $$b_{0}=\frac{p-2q}{pq},\;b_{1}=-\frac{p+2q}{p-2q}\;\mathrm{and}\;b_{2}=\frac{p+2q}{2p-q}.$$

With equations (3.16) and (3.17), we derive the following linear equations for the two equilibrium paths:

3.18 $$\theta_{m}+\frac{1-\tan^{2}(\pi /8)}{1+\tan^{2}(\pi /8)}\theta_{s}=0\;\mathrm{and}\;\theta_{m}-\frac{1+2\tan(\pi /8)}{1-2\tan(\pi /8)}\theta_{s}+\frac{1+2\tan(\pi /8)}{2-\tan(\pi /8)}=0,$$

or

3.19 $$\theta_{s}=-\sqrt{2}\theta_{m}\;\mathrm{and}\;\theta_{s}=\frac{-5+4\sqrt{2}}{7}\theta_{m}+\frac{5-3\sqrt{2}}{7}.$$

### 3.3. Initial stiffness and load–displacement behaviours

The tangent stiffness of the structural system is expressible as

3.20 $$\frac{d{\tilde{F}}_{2}}{d{\tilde{d}}_{2}}=\frac{\partial {\tilde{F}}_{2}}{\partial \phi }\frac{d\phi }{d{\tilde{d}}_{2}}+\frac{\partial {\tilde{F}}_{2}}{\partial \psi }\frac{d\psi }{d{\tilde{d}}_{2}}.$$

We first derive the initial stiffness of the simplified system for ∀*κ* from equation (3.20). Using equations (3.2) and (3.3), equation (2.14) can be rewritten as

3.21 $$\begin{array}{rl} \frac{\partial \tilde{\Pi }}{\partial \phi } & =\kappa \phi +(\phi -\psi )-{\tilde{F}}_{2}(-4p\phi -4q)=0\\ \;\text{and}\;\frac{\partial \tilde{\Pi }}{\partial \psi } & =\kappa \psi -(\phi -\psi )-{\tilde{F}}_{2}(-2q\psi +2p)=0 \end{array}\}$$

and rearranged to give

3.22 $${\tilde{F}}_{2}=\frac{\kappa \phi +(\phi -\psi )}{-4p\phi -4q}\;\mathrm{and}\;{\tilde{F}}_{2}=\frac{\kappa \psi -(\phi -\psi )}{-2q\psi +2p}.$$

The partial derivatives of the first relation in equation (3.22) with respect to *ϕ* and *ψ* are

3.23 $$\frac{\partial {\tilde{F}}_{2}}{\partial \phi }=\frac{\kappa +1}{-4p\phi -4q}+\frac{4p(\kappa \phi +\phi -\psi )}{{\left(-4p\phi -4q\right)}^{2}}\;\mathrm{and}\;\frac{\partial {\tilde{F}}_{2}}{\partial \psi }=\frac{1}{4p\phi +4q}.$$

In equation (3.1), the dimensionless displacement ${\tilde{d}}_{2}$ of a unit cell corresponds to

3.24 $${\tilde{d}}_{2}=-2p\phi^{2}-4q\phi -q\psi^{2}+2p\psi .$$

Hence, the ordinary derivatives of equation (3.24) with respect to *ϕ* and *ψ* can be expressed by

3.25 $$\frac{d{\tilde{d}}_{2}}{d\phi }=-4(p\phi +q)-2(q\psi -p)\frac{d\psi }{d\phi }$$

and

3.26 $$\frac{d{\tilde{d}}_{2}}{d\psi }=-4(p\phi +q)\frac{d\phi }{d\psi }-2(q\psi -p).$$

Note that the derivative *dϕ*/*dψ* in equation (3.25) or (3.26) can be derived from the condition g*=0 in equation (3.4). Thus

3.27 $$\frac{d\phi }{d\psi }=\frac{\{\kappa (2p-q)+2p+q\}\phi -2q\psi +2\kappa q-p+2q}{4p\phi +\{\kappa (-2p+q)-p-q\}\psi -\kappa p-p+2q}.$$

Substituting equations (3.23) and (3.25)–(3.27) into equation (3.20) yields the initial stiffness ${\tilde{K}}_{0}$ of the simplified system,

3.28 $$\begin{array}{rl} {\tilde{K}}_{0} & =\lim_{\phi ,\psi \to 0}\frac{d{\tilde{F}}_{2}}{d{\tilde{d}}_{2}}\\ & =\lim_{\phi ,\psi \to 0}\left(\frac{\partial {\tilde{F}}_{2}}{\partial \phi }\frac{d\phi }{d{\tilde{d}}_{2}}+\frac{\partial {\tilde{F}}_{2}}{\partial \psi }\frac{d\psi }{d{\tilde{d}}_{2}}\right)\\ & =\frac{\kappa (\kappa +2)}{4\{(p^{2}+4q^{2})\kappa +{\left(p-2q\right)}^{2}\}}. \end{array}$$

Obviously, ${\tilde{K}}_{0}$ in equation (3.28) also corresponds to the initial stiffness of the structural system without approximation.

**Figure 8.**
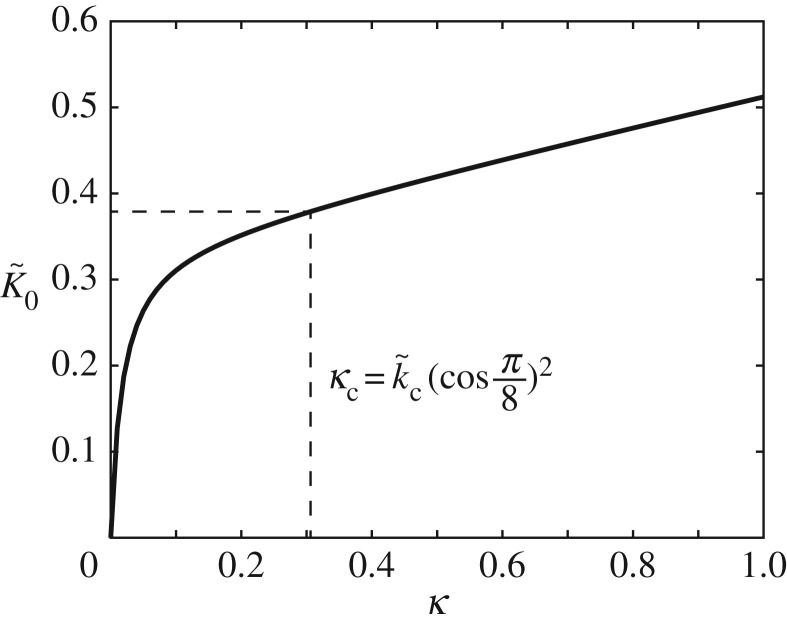
Relationship between overall initial stiffness and internal stiffness (${\tilde{K}}_{0}$ versus *κ*).

Using equation (3.28), the plot of ${\tilde{K}}_{0}$ versus *κ* (figure 8) shows that, as *κ* increases, the slope diminishes and approaches a constant asymptotically. Indeed, the limit of ${\tilde{K}}_{0}$ as *κ* approaches zero or infinity can be calculated. We have

3.29 $$\lim_{\kappa \to 0}\frac{d{\tilde{K}}_{0}}{d\kappa }=\frac{1}{2{\left(p-2q\right)}^{2}}\approx 19.8995$$

and

3.30 $$\lim_{\kappa \to \infty }\frac{d{\tilde{K}}_{0}}{d\kappa }=\frac{1}{4(p^{2}+4q^{2})}\approx 0.1737.$$

Because κ→∞ either as ℓ→∞ or r→0, the transformation is dominated as *κ* increases by the distortion of the linked square, which is characterized by the structural behaviour in terms of *θ*_s_ (figure 7*c*). That is, the system turns out to be conventional as κ→∞ because there is a linear relationship between the overall initial stiffness and the internal stiffness.

We next investigate the load–displacement curve of the simplified system under the transition state. Considering the primary path given by equation (3.14), we can represent *ϕ* and *ψ* as functions of *θ*_s_,

3.31 $$\phi =\theta_{m}+\theta_{s}=(-p^{2}+q^{2}+1)\theta_{s}=2q^{2}\theta_{s}$$

and

3.32 $$\psi =\theta_{m}-\theta_{s}=(-p^{2}+q^{2}-1)\theta_{s}=-2p^{2}\theta_{s}.$$

Substituting equations (3.31) and (3.32) into the first relation in equation (3.22), we find

3.33 $${\tilde{F}}_{2}=-\frac{(q^{2}\kappa +1)\theta_{s}}{4pq^{2}\theta_{s}+2q}\;\Leftrightarrow \;\theta_{s}=-\frac{2q{\tilde{F}}_{2}}{4pq^{2}{\tilde{F}}_{2}+\kappa q^{2}+1}.$$

Recalling that the critical point is *κ*_c_=(*p*−2*q*)/{*pq*(2*p*−*q*)}, equation (3.33) becomes

3.34 $$\theta_{s}=-\frac{{\tilde{F}}_{2}}{2pq{\tilde{F}}_{2}+C},$$

where

3.35 $$C=\frac{p^{2}-q^{2}}{pq(2p-q)}.$$

We also substitute equations (3.31) and (3.32) into equation (3.24) to obtain

3.36 $$\begin{array}{rl} {\tilde{d}}_{2} & =(-8pq^{4}-4p^{4}q)\theta_{s}^{2}+(-8q^{3}-4p^{3})\theta_{s}\\ & =-4(p^{3}+2q^{3})(pq\theta_{s}+1)\theta_{s}. \end{array}$$

With equations (3.34)–(3.36), we derive the dimensionless load–displacement relation on the primary equilibrium path,

3.37 $${\tilde{d}}_{2}=\frac{4(p^{3}+2q^{3}){\tilde{F}}_{2}(pq{\tilde{F}}_{2}+C)}{{\left(2pq{\tilde{F}}_{2}+C\right)}^{2}},$$

for which there are the two types of limit, one for infinite tensile loading and the other for infimum compressive loading; that is,

3.38 $$\begin{array}{rl} {\tilde{d}}_{2} & \to \frac{p^{3}+2q^{3}}{p^{2}q^{2}},\;\mathrm{as}\;{\tilde{F}}_{2}\to \infty \\ \;\text{and}\;{\tilde{d}}_{2} & \to -\infty ,\;\mathrm{as}\;{\tilde{F}}_{2}\to -\frac{C}{2pq}+0. \end{array}\}$$

We hereafter express the infimum load as ${\tilde{F}}_{\inf}\equiv -C/2pq$. Note that equation (3.37) has another isolated load–displacement curve beyond ${\tilde{F}}_{\inf}$, which implies that the compressively loaded structure for ${\tilde{F}}_{\inf}<0$ is reversed if the bar-to-bar contact is neglected, and it becomes stable if subjected to an effective tensile load. The system stability will be discussed in the next subsection.

Similarly, we consider the secondary equilibrium path of *θ*_m_+*b*_1_*θ*_s_+*b*_2_=0 in equation (3.16). In this case, the dependence of *ϕ* and *ψ* on *θ*_s_ is

3.39 $$\phi =\theta_{m}+\theta_{s}=\frac{2p}{p-2q}\theta_{s}-\frac{p+2q}{2p-q}$$

and

3.40 $$\psi =\theta_{m}-\theta_{s}=\frac{4q}{p-2q}\theta_{s}-\frac{p+2q}{2p-q}.$$

On substituting these equations into the first identity in equation (3.22), we obtain as a consequence of *θ*_s_ vanishing the following constant force:

3.41 $$\begin{array}{rl} {\tilde{F}}_{2} & =\frac{\frac{2p(p+2q)(p-2q)}{q}\theta_{s}-\frac{(p+2q){\left(p-2q\right)}^{2}}{pq(2p-q)}}{-8p^{2}(2p-q)\theta_{s}+4(p-2q)}\\ & =-\frac{\left(p+2q)(p-2q\right)}{4pq(2p-q)}\\ & \equiv {\tilde{F}}_{c}. \end{array}$$

Numerically, the constant critical force ${\tilde{F}}_{c}$ is

$$\begin{array}{rl} {\tilde{F}}_{c} & =-\frac{1-4\tan^{2}(\pi /8)}{4\sin(\pi /8)\left(2-\tan(\pi /8)\right)}\\ & \approx -0.1292. \end{array}$$

The result obtained means that, after switching from the primary to the secondary path via the critical point, the simplified system maintains the same constant level of force regardless of whether it transforms to Pattern-D or -S.

**Figure 9.**
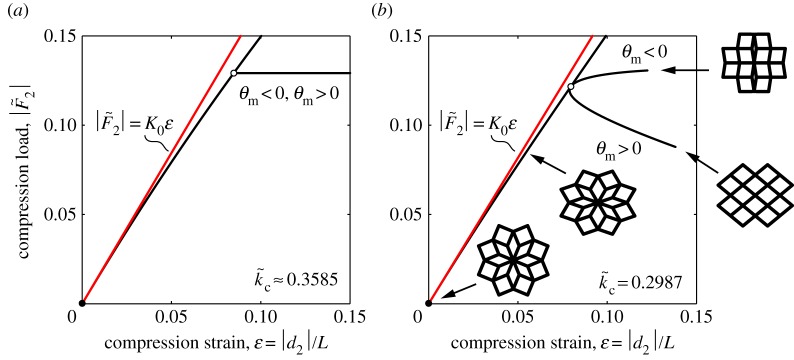
(*a*) Compression load–strain curves of the simplified system under the transition state represented by equations (3.37) and (3.41); (*b*) numerical compression load–strain curves of the structural system under the transition state ($\tilde{k}=0.2987$); the series of insets shows snapshots of the transformed unit cell. In both figures, the red lines extrapolate the slope of the initial stiffness.

Figure 9*a* shows the compression load–strain relation of the simplified system, based on equations (3.37) and (3.41). The compression strain is calculated by $\varepsilon =|d_{2}|/L=|{\tilde{d}}_{2}|\ell /L$. The slope of the initial stiffness $K_{0}=({\tilde{K}}_{0}L)/\ell$ is also superposed in figure 9*a*, indicated by a red line. As shown in figure 9*b*, we added the compression load–strain relation of the structural system, which was numerically analysed. In comparing figure 9*a*,*b*, we find that, in the structural system, the secondary path towards Pattern-D (*θ*_m_>0) exhibits unstable elastic deformation, i.e. the compression load starts to decrease via the critical point, whereas the other path towards Pattern-S (*θ*_m_<0) is stable. Such an unstable behaviour is not observed in the simplified system, which maintains a constant force after switching. We also find that the primary path of the simplified system tends to depart from the slope of the initial stiffness in comparison with that of the structural system. In the next subsection, we discuss the stability of the equilibrium paths in the simplified system under the transition state.

### 3.4. Stability of the transition state

We consider the following Hessian matrix:

3.42 $$H=\left(\begin{array}{cc} \frac{\partial^{2}\tilde{\Pi }}{\partial \phi^{2}} & \frac{\partial^{2}\tilde{\Pi }}{\partial \phi \partial \psi }\\ \frac{\partial^{2}\tilde{\Pi }}{\partial \psi \partial \phi } & \frac{\partial^{2}\tilde{\Pi }}{\partial \psi^{2}} \end{array}\right).$$

For the total potential energy $\tilde{\Pi }$ in equation (3.1), each component of ***H*** becomes

$$\frac{\partial^{2}\tilde{\Pi }}{\partial \phi^{2}}=\kappa +4p{\tilde{F}}_{2}+1,\;\frac{\partial^{2}\tilde{\Pi }}{\partial \psi^{2}}=\kappa +2q{\tilde{F}}_{2}+1,\;\frac{\partial^{2}\tilde{\Pi }}{\partial \phi \partial \psi }=\frac{\partial^{2}\tilde{\Pi }}{\partial \psi \partial \phi }=-1.$$

Then, the determinant of ***H*** is expressed as

3.43 $$\det\;H=8pq{\tilde{F}}_{2}^{2}+2(2p+q)(\kappa +1){\tilde{F}}_{2}+\kappa (\kappa +2).$$

When *κ*=*κ*_c_, equation (3.43) can be factored as

3.44 $$\det H=8pq({\tilde{F}}_{2}-{\tilde{F}}_{\inf})({\tilde{F}}_{2}-{\tilde{F}}_{c}).$$

A derivation of this factorization is described in appendix B.

**Figure 10.**
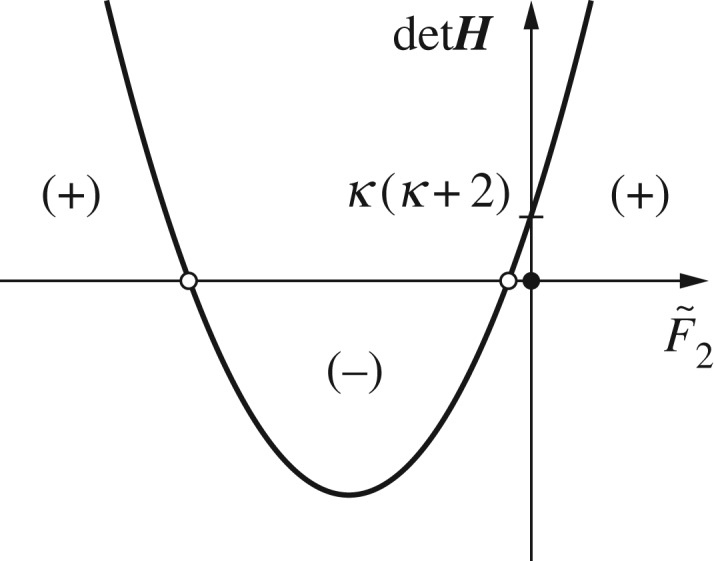
Schematic of the quadratic curve of det***H*** versus ${\tilde{F}}_{2}$ obtained using equation (3.44).

Figure 10 presents a schematic of the quadratic curve for det***H*** as a function of ${\tilde{F}}_{2}$, indicating that the system under the transition state is unstable within the two crossing points with det***H***=0, which are the two singular forces, ${\tilde{F}}_{\inf}$ and ${\tilde{F}}_{c}$, given in equations (3.38) and (3.41). Hence, the system stability criteria obtain

3.45 $$\det\;H(\kappa_{c},{\tilde{F}}_{2})\left\{ \begin{array}{ll} >0 & \mathrm{for}\;{\tilde{F}}_{2}\in (-\infty ,{\tilde{F}}_{\inf}),\\ \leq 0 & \mathrm{for}\;{\tilde{F}}_{2}\in [{\tilde{F}}_{\inf},{\tilde{F}}_{c}],\\ >0 & \mathrm{for}\;{\tilde{F}}_{2}\in ({\tilde{F}}_{c},\infty ). \end{array}\right.$$

## 4. Summary and conclusion

We clarified the static transformation of a certain periodic bar-and-joint framework under uniaxial loading with two angular degrees of freedom (*θ*_m_,*θ*_s_) describing the different types of component motions, i.e. rotation and distortion of a linked square. Performing a structural stability analysis of the system, we revealed the transition mechanism whereby the structure forms into either a diamond- or a square-patterned configuration referred to as Pattern-D (*θ*_m_>0) and Pattern-S (*θ*_m_<0).

We first determined the total potential energy of the structural unit composed of identical bars and joints connected with linear and rotational springs. Exploring the equilibrium paths of the dimensionless structural system, we found that the loaded structure has a transition state for a critical internal stiffness, in which the primary equilibrium path bifurcates into two secondary paths towards Pattern-D or -S. The structure with internal stiffness below the critical value undergoes Pattern-S, otherwise it undergoes Pattern-D. We explained these transition behaviours through changes in the hyperplane of a hyperbolic paraboloid (g*-function in equation (2.19)) for when g*=0.

To determine the basic mechanism of the structural system, we derived the exact solution of the transition state modelled using a weak nonlinear model. As a result, we proved that this simplified system also exhibits a similar transition state, where the critical point corresponds to a saddle point of the g*-function, and we described both equilibrium paths using two concise linear equations depending on the two state angles. In addition, we obtained the load–displacement curves of the simplified system under the transition state, which in particular exhibits a constant compression load along the secondary path regardless of whether the system undergoes Pattern-D or -S. Last, we discussed the stability of the simplified system under the transition state and showed that the system is unstable when the applied compression force ranges between the critical and infimum forces (${\tilde{F}}_{2}\in [{\tilde{F}}_{\inf},{\tilde{F}}_{c}]$).

The modelling of the simplified system can be generalized for other initial configurations of the proposed structure because we only used the identity *p*^2^+*q*^2^=1 for the development of all these equations in §3. Specifically, we parametrized setting p=cos⁡α and q=sin⁡α, where *α* determines its initial configuration formed by the rotational operation of Pattern-D or -S. It might be said that such a transition mechanism is broadly applicable to subtypes of the structures and the abstract framework.

We also assessed the physical nature of the internal stiffness *κ*. From the dimensionless parameter in (2.6), we have $\kappa =\tilde{k}p^{2}=(kp^{2}\ell^{2})/(16r)$, which means that we can control *κ* with several variables. For example, *κ* becomes larger when ℓ increases and the other variables are constant. Hence, the expectation is that a growing system under some environmental loads has a similar transition state, as discussed in appendix A. Apart from the elastic problems presented, we could create structural frameworks with damping members and extend them to three-dimensional structures with multiple degrees of freedom as encountered in viscoelastic, dynamic and multiple-body problems. What develops from these transition systems will result in good mechanical and optical devices equipped with a bi-stiffness property [55,56] and switchable optical characteristics [62], which are able to operate according to the internal and/or external changes.

## Figures and Tables

**Figure 11.**
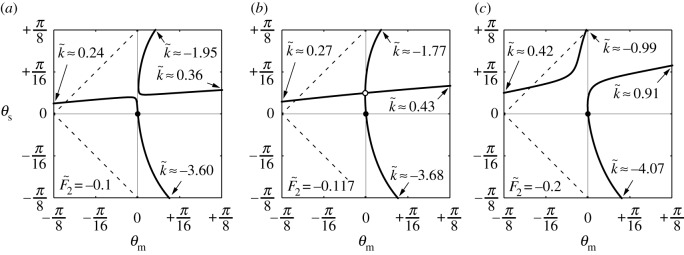
Calculated equilibrium paths (*θ*_m_,*θ*_s_) of the structural system with three different ${\tilde{F}}_{2}$ values: (*a*) ${\tilde{F}}_{2}=-0.1$, (*b*) ${\tilde{F}}_{2}=-0.117$ and (*c*) ${\tilde{F}}_{2}=-0.2$.

## Data accessibility

This paper has no experimental data.

## Appendix A. Relationship between the internal stiffness and system transformation under a constant compression

We here discuss how the system, subjected to a fixed load, behaves as the internal stiffness is changed. In a similar manner as in §(c), we obtain the following relationship by eliminating $\tilde{k}$ in equation (2.14):

$$(S_{\phi }+S_{\psi })(\phi -\psi )-{\tilde{F}}_{2}\left(S_{\psi }\frac{\partial {\tilde{d}}_{2}}{\partial \phi }-S_{\phi }\frac{\partial {\tilde{d}}_{2}}{\partial \psi }\right)=0.$$

Thus

A 1 $$(S_{\phi }+S_{\psi })(\phi -\psi )+2{\tilde{F}}_{2}\left\{2S_{\psi }\sin\left(\frac{\pi }{8}+\phi \right)+S_{\phi }\cos\left(\frac{\pi }{8}+\psi \right)\right\}=0.$$

Let *h** be the function on the LHS of equation (A.1). We investigate the equilibrium path of the structural system to solve the problem: find (*ϕ*,*ψ*)∈*Ω** such that $h^{*}(\phi ,\psi ,{\tilde{F}}_{2})=0$ for a fixed ${\tilde{F}}_{2}\in R$.

Figure 11*a*–*c* shows the calculated state diagrams of the structural system for ${\tilde{F}}_{2}=-0.1$, ${\tilde{F}}_{2}=-0.117$ and ${\tilde{F}}_{2}=-0.2$, respectively. The structural system under a critical compression is seen to have a transition state induced by controlling the internal stiffness.

## Appendix B. Factorization of the determinant of the Hessian matrix

Substituting the formulae for ${\tilde{F}}_{\inf}$ and ${\tilde{F}}_{c}$ into equation (3.44), we find the following quadratic function:

B 1 $$\begin{array}{rl} \det H & =8pq({\tilde{F}}_{2}-{\tilde{F}}_{\inf})({\tilde{F}}_{2}-{\tilde{F}}_{c})\\ & =8pq\left\{{\tilde{F}}_{2}+\frac{p^{2}-q^{2}}{2p^{2}q^{2}(2p-q)}\right\}\left\{{\tilde{F}}_{2}+\frac{p^{2}-4q^{2}}{4pq(2p-q)}\right\}\\ & =8pq{\tilde{F}}_{2}^{2}+A{\tilde{F}}_{2}+B, \end{array}$$

where each of the two coefficients, *A* and *B*, can be calculated as

$$\begin{array}{rl} A & =8pq\cdot \frac{2(p^{2}-q^{2})+pq(p^{2}-4q^{2})}{4p^{2}q^{2}(2p-q)}\\ & =\frac{4p^{2}-4q^{2}+2pq(p^{2}-4q^{2})}{pq(2p-q)}\\ & =\frac{4p^{2}-4q^{2}+2pq(4p^{2}-q^{2}-3)}{pq(2p-q)}\\ & =2(2p+q)\cdot \frac{p-2q+pq(2p-q)}{pq(2p-q)}\\ \;\mathrm{and}\;\;B & =8pq\cdot \frac{\left(p^{2}-q^{2})(p^{2}-4q^{2}\right)}{8p^{3}q^{3}{\left(2p-q\right)}^{2}}\\ & =\frac{\left(p-2q)(p^{3}+2p^{2}q-pq^{2}-2q^{3}\right)}{p^{2}q^{2}{\left(2p-q\right)}^{2}}\\ & =\frac{\left(p-2q)\{p(1-q^{2})+2p^{2}q-pq^{2}-2(1-p^{2})q\right\}}{p^{2}q^{2}{\left(2p-q\right)}^{2}}\\ & =\frac{\left(p-2q)\{(p-2q)+2pq(2p-q)\right\}}{p^{2}q^{2}{\left(2p-q\right)}^{2}}. \end{array}$$

Clearly, equation (B 1) is consistent with equation (3.43) with *κ*=*κ*_c_.

## Appendix C. Nomenclature

*a*,…,*e*: parameters of a conic section
*C*: constant of (*p*^2^−*q*^2^)/{*pq*(2*p*−*q*)} as defined in equation (3.35)
***d***: displacement vector (*d*_1_ *d*_2_)^*T*^ for a unit cell
*f*: sum of non-dimensional potential energies regarding two types of springs
***F***: force vector (*F*_1_ *F*_2_)^*T*^ acting on a unit cell
${\tilde{F}}_{c}$: critical force of a simplified system as defined in equation (3.41)
${\tilde{F}}_{\inf}$: infimum force (−*C*/2*pq*) of a simplified system under a transition state
*g*: function of the LHS of equation (2.15)
*g**: function defined by $(1/2)(\partial {\tilde{d}}_{2}/\partial \phi )(\partial {\tilde{d}}_{2}/\partial \psi )g$ as in equation (2.19)
*h**: function of the LHS of equation (A.1)
***H***: Hessian matrix of $\tilde{\Pi }(\phi ,\psi )$
*k*: constant of a linear half-length spring binding neighbouring unit cells
${\tilde{k}}_{c}$: critical value of $\tilde{k}$ as a system has a transition state
${\tilde{K}}_{0}$: dimensionless initial stiffness of a system
ℓ: bar length
*L*: cell length
*p*,*q*: mnemonic symbols defined by p=cos⁡(π/8) and q=sin⁡(π/8)
R: set of real numbers
$R_{+}$: set of positive reals
*S*_*ϕ*_,*S*_*ψ*_: mnemonic symbols defined by $S_{\phi }={\tilde{u}}_{1}(\partial {\tilde{u}}_{1}/\partial \phi )$ and $S_{\psi }={\tilde{u}}_{2}(\partial {\tilde{u}}_{2}/\partial \psi )$
***u***: elongation vector of (*u*_1_ *u*_2_)^*T*^ for horizontal and vertical linear springs
*U*_*k*_: potential energy of linear springs
*U*_*r*_: potential energy of rotational springs
*W*: work done by ***F***
(*x*_1_,*x*_2_): two-dimensional orthogonal coordinate system
*ε*: compression strain in a unit cell
*θ*_m_: one of two state angles, representing the rotational paths of Pattern-D or -S
*θ*_s_: one of two state angles, representing the rhombic distortion of linked squares
*κ*: dimensionless parameter ($\kappa =\tilde{k}p^{2}$) of a linear spring
*κ*_c_: a critical value of *κ* as a system has a transition state
*Π*: total potential energy of a system
*Ω*: parameter space of *θ*_m_ and *θ*_s_
∂*Ω*: boundary of domain *Ω*, which indicates bar-to-bar contact
*Ω**: parameter space of *ϕ* and *ψ*
*ϕ*, *ψ*: converted parameters of two state angles (*θ*_m_,*θ*_s_) as defined in equation (2.7)
$\tilde{∙}$: dimensionless parameter of •; e.g. see equation (2.6)
