# Correction: ‘Transition mechanism for a periodic bar-and-joint framework with limited degrees of freedom controlled by uniaxial load and internal stiffness’ (2018), by Tanaka *et al.*

**DOI:** 10.1098/rsos.231893

**Published:** 2024-01-10

**Authors:** H. Tanaka, K. Hamada, Y. Shibutani

*R. Soc. Open Sci.* **5**: 180139 (Published online 13 June 2018). (https://doi.org/10.1098/rsos.180139)

The original version of this article contains the error in equation (2.2) as

2.2 $$d=\left(\begin{array}{c} d_{1}\\ d_{2} \end{array}\right)=\left(\begin{array}{c} 2\ell \left\{\cos\left(\frac{\pi }{8}+\theta_{m}-\theta_{s}\right)+\sin\left(\frac{\pi }{8}+\theta_{m}+\theta_{s}\right)-2\cos\frac{\pi }{8}-\sin\frac{\pi }{8}\right\}\\ 2\ell \left\{\cos\left(\frac{\pi }{8}+\theta_{m}+\theta_{s}\right)+\sin\left(\frac{\pi }{8}+\theta_{m}-\theta_{s}\right)-2\cos\frac{\pi }{8}-\sin\frac{\pi }{8}\right\} \end{array}\right).$$

This should be corrected as

2.2 $$d=\left(\begin{array}{c} d_{1}\\ d_{2} \end{array}\right)=\left(\begin{array}{c} 2\ell \left\{2\cos\left(\frac{\pi }{8}+\theta_{m}-\theta_{s}\right)+\sin\left(\frac{\pi }{8}+\theta_{m}+\theta_{s}\right)-2\cos\frac{\pi }{8}-\sin\frac{\pi }{8}\right\}\\ 2\ell \left\{2\cos\left(\frac{\pi }{8}+\theta_{m}+\theta_{s}\right)+\sin\left(\frac{\pi }{8}+\theta_{m}-\theta_{s}\right)-2\cos\frac{\pi }{8}-\sin\frac{\pi }{8}\right\} \end{array}\right).$$

The corrected equation (2.2) above is used in the subsequent calculations in the original version of this article.

